# Zoonotic enteric parasites in Mongolian people, animals, and the environment: Using One Health to address shared pathogens

**DOI:** 10.1371/journal.pntd.0009543

**Published:** 2021-07-08

**Authors:** Amber N. Barnes, Anu Davaasuren, Uyanga Baasandavga, Paul M. Lantos, Battsetseg Gonchigoo, Gregory C. Gray

**Affiliations:** 1 Institute of Veterinary Medicine, Ulaanbaatar, Mongolia; 2 School of Medicine, Duke University, Durham, North Carolina, United States of America; 3 Department of Public Health, University of North Florida, Jacksonville, Florida, United States of America; 4 National Center for Communicable Disease, Ulaanbaatar, Mongolia; 5 National Center for Zoonotic Disease, Ulaanbaatar, Mongolia; 6 Duke Global Health Institute, Duke University, Durham, North Carolina, United States of America; 7 Global Health Research Institute, Duke-Kunshan University, Kunshan, Jiangsu, China; 8 Emerging Infectious Diseases Program, Duke-NUS Medical School, Singapore; Emory University, UNITED STATES

## Abstract

**Background:**

*Cryptosporidium spp*. and *Giardia duodenalis* are important zoonotic enteric pathogens of One Health concern for humans, animals, and the environment. For this study, we investigated parasite prevalence and risk factors among rural, peri-urban, and urban households and environments of Mongolia.

**Methods:**

This cross-sectional study implemented a household risk factor survey at 250 home sites along with sample collection from humans, animals, flies, and drinking water. Multiplex real-time PCR analysis was conducted to look for *Cryptosporidium spp*. and/or *Giardia duodenalis* within household samples.

**Results:**

Lab analysis found one or both zoonotic parasites at 20% of the participating households (51/250). Human samples had a parasite prevalence of 6.4% (27/419), domestic animals at 3.3% (19/570), pooled filth flies at 14.8% (17/115), and drinking water samples at 2% (5/250). Parasite presence at the household was significantly associated with a household’s use of an improved drinking water source (OR 0.27; CI 0.12–0.61; p = < 0.01), having an indoor handwashing site (OR 0.41; CI 0.19–0.92; p = 0.03), domestic animal ownership (OR 2.40; CI 1.02–5.65; p = 0.05), and rural location (OR 0.50; CI 0.25–0.98; p = 0.04). Household use of an improved drinking water source remained significant in the multivariate model (OR 0.16; CI 0.04–0.68; p = 0.01).

**Conclusion:**

In Mongolia, public and veterinary health are intertwined, particularly for rural herding households. Increased access to safe water, sanitation and hygiene infrastructure could help prevent further transmission of zoonotic enteric parasites. Public health interventions, policy and messaging should utilize a One Health framework employing joint leadership from local human and animal health sectors.

## Author summary

The zoonotic enteric parasites of *Cryptosporidium spp*. and *Giardia duodenalis* are a One Health concern for human, animal and environmental health. In this study, human, animal, fly, and water samples were collected from 250 households in rural, peri-urban and urban areas of Mongolia in conjunction with a household risk factor survey. Twenty percent of the households (n = 51) had at least one sample positive for *Cryptosporidium spp*. and/or *Giardia duodenalis* with several households demonstrating parasite presence in multiple sample types (9.8%, 5/51). Households that used unimproved drinking water, did not have access to an indoor handwashing site, were located in rural areas, and owned animals were associated with higher rates of parasite presence. Our findings show that zoonotic enteric parasite exposure is occurring at the household level and from different hosts, vectors, and vehicles. Public health efforts to prevent the further spread of disease should concentrate on increased water, sanitation and hygiene measures and One Health collaboration between human and animal health care disciplines.

## Introduction

The gastrointestinal zoonotic parasites of *Cryptosporidium spp*. and *Giardia duodenalis* cause a significant portion of the diarrheal disease burden worldwide [1–2]. Between the years 2011 and 2016, *Cryptosporidium spp*. are estimated to have caused 63% of the reported global outbreaks of waterborne protozoal parasites while *Giardia spp*. were responsible for approximately 37% [3]. These pathogens have proven to be important etiological agents for childhood enteric disease, particularly among those living in households and communities that lack safe water, sanitation and hygiene [1,3–7]. Moderate to severe diarrheal disease from parasites such as *Cryptosporidium spp*. can increase a child’s risk of death by 8.5 to 12 times that of a child without diarrhea and limit their nutritional intake leading to stunted physical growth [1,5]. These protozoan parasites can be transmitted through contact with contaminated water, surfaces and fomites, filth flies and unwashed hands [2,8–12]. These pathogens also serve as a source of global foodborne disease [13–15].

*Cryptosporidium spp*. and *Giardia duodenalis* are resilient zoonotic enteric parasites (ZEPS) that can survive outside of a host from hours to months on surfaces, in soil, and in raw and potable water, regardless of chlorination [8,14,16–18]. The ownership and presence of domestic animals in close proximity to households presents an exposure risk for accidental fecal-oral ingestion from contact with unmanaged animal waste, whether directly through the collection of animal dung for cooking fuel or indirectly from drinking water from a contaminated source [6,19–20]. Wildlife, livestock, companion animals like dogs and cats, and synanthropic rodents can all serve as disease hosts and can spread these pathogens to one another, to people, and into their environment making these zoonotic agents a true One Health challenge [21–24].

In Mongolia, the health of humans, animals, and the environment have been intertwined for millennia through the tradition of nomadic herding [25]. Although the nation has the second lowest population density in the world with slightly more three million people, it is home to more than 66 million livestock [26–27]. Pastoralism and herding remain a popular livelihood and cultural practice within Mongolia, yet also present a unique threat for enteric zoonoses [28–32]. Close contact with free-grazing livestock occurs frequently at the household level as the animals are used for meat and milk, racing and herding, sale and trade, and the manufacture of animal goods such as leather and fiber [29,33–35]. The nomadic herding lifestyle often lead to interactions between domestic animals and wildlife across their many shared environments, particularly pasture and water sources, thus creating a risk for intraspecies transmission [36,37]. Endemic zoonotic diseases such as brucellosis, echinococcosis, plague, anthrax, and rabies are common and recent years have seen a growth in emerging and reemerging diseases of public health importance (e.g. Seoul hantavirus) [37–38]. However, diarrheal diseases are often unreported and undiagnosed in Mongolia, despite accounting for over 13% of all reported infectious disease [39]. Among hospitalized diarrheal patients in the capital city of Ulaanbaatar, the prevalence of *Cryptosporidium spp*. and *Giardia duodenalis* have been reported to be 3.6% and 5.1%, respectively [40]. In neighboring China, research has demonstrated an average prevalence of 2.97% for *Cryptosporidium spp*. and a range of 0.85% to 9.46% for *Giardia spp*. among patients [41–43]

Access to safe water, sanitation and hygiene services varies across the country with a stark divide between urban housing complexes and rural, nomadic, or off-grid *ger* (yurt) households [44]. Indiscriminate human and animal waste can be found in rural and peri-urban areas that lack sanitation and waste management systems designed to safely remove, store, and/or treat effluent. In addition, without safe water for drinking, cooking, and personal hygiene measures, it can be difficult to reduce the risk for enteric pathogen exposures in the household. In remote areas, seasonal drinking water sources are shared between animals and humans leading to collective exposure risks for ZEP transmission through contaminated water [35].

Understanding more about the ecology and epidemiology of zoonotic enteric parasites can help guide collaborative efforts between human and animal health care providers to prevent disease exposure and transmission. The purpose of this One Health study was to determine the prevalence of either *Cryptosporidium spp*. or *Giardia spp*. in humans, animals, and the environment of Mongolia and to identify household risk factors associated with pathogen presence.

## Methods

### Ethical approval

Approval for this study was granted through the ethics committees of Duke University [Protocol ID: PRO 00076868] and the Mongolian Ministry of Health. This study was exempt under the Institutional Animal Care and Use Committee (IACUC).

### Study setting and design

**Fig 1:**
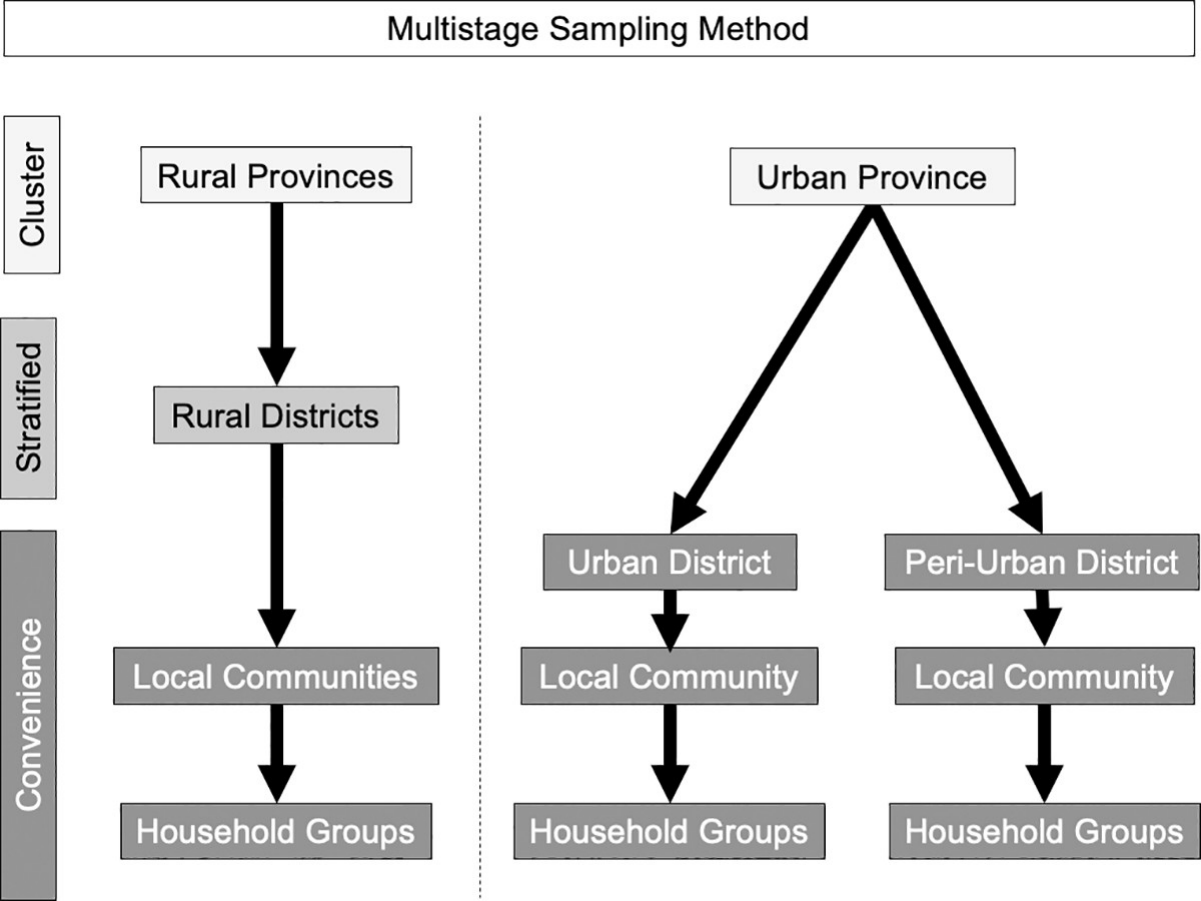
Sample strategy for household selection in rural, peri-urban and urban study sites of Mongolia.

This cross-sectional study was conducted between April and October 2017 across four Mongolian provinces (*aimags)*: Tov, Selenge, Zavkhan, and Dundgobi. The prevalence of *Cryptosporidium spp*. in the stool of individual gastrointestinal patients was estimated at 5% in a previous work conducted in Mongolia and was assumed a true prevalence and used to calculate a sample size for this study using OpenEpi Version 3 (www.OpenEpi.com) [40]. The open source calculator indicated at least 141 people would need to be sampled for 95% confidence intervals using a +/-3.6% prevalence window with 80% power. A total of 250 households participated in this study with 50 households at each site: urban apartments and a peri-urban ger district of the capital city Ulaanbaatar in Tov province and herding households among the rural provinces of Selenge, Zavkhan, and Dundgobi. Survey administration was seasonally divided with half of the sampling occurring from April through June and the other half occurring from August through October. A multistage sampling strategy was used to determine geographic clusters, determine the sampling district and community, and finally select participating households (Fig 1). Each participating household provided samples of drinking water, animal stool (if present), human stool, flies (if present), geographic location and household survey answers related to risk factors for zoonotic enteric parasite transmission during either the first or second sampling season.

Provinces were selected based on provincial governmental data on livestock density and human-animal interactions, representation of various ecosystems, a range of population demographics and household characteristics, and ability to safely travel to home sites. In rural provinces, animal herding is the primary occupation and human-animal contact occurs daily. However the ecology of the provinces varies greatly. The northern province of Selenge has taiga, forest, and mixed forest-grassland steppe while the western province of Zavkhan is situated in mountain steppe, the Gobi desert, and a large lake basin [45,46]. Dundgobi province is centrally located within the country and is made up of semi-arid steppe and low height hills. It is located between the desert and steppe areas [45,46].

Within the rural province sampling clusters, districts (*soums)*, were ranked by highest to lowest animal density and one district was selected randomly from the highest 25% listed (Tsagaansuur in Selenge province, Tosontsengel in Zavkhan province, and Erdenedalai in Dundgovi province). Smaller administrative units under the district designation are rural communities (*baghs)*. Using the same animal density ranking method and in consultation with local veterinarians on weather, safety and vehicle accessibility to herding households, two rural community locations were chosen within each district. At the rural community level, even smaller herding household groups known as *khot ails* are located across the landscape by geography (ex. near water or in the mountains). Using a convenience sampling strategy, local guides assisted the field team in transecting the rural community area to look for household groups based on seasonal ger movement. These groups may have one household or multiple households. When a household group was found, all ger households with separate livestock were invited to participate.

Within the urban Tov province, there are 27 *soum* districts and nine municipal districts in and around the nation’s capital city, Ulaanbaatar. Ulaanbaatar is home to approximately 1.5 million people, with peri-urban ger settlements growing each year along with the urban poverty rate [26,47]. These informal communities are typically not included in the urban infrastructure of water, sanitation, or household heating [48,49]. Many of these households practice animal husbandry for food and income similar to their rural counterparts [50]. Outside of the capital city, Tov province is primarily forest-steppe or steppe landscape [45,46]. Ulaanbaatar was chosen as a study cluster site with urban sampling in the Bayangol district and peri-urban ger households sampled in the Bayanzurkh district. Urban Bayangol respondents were invited to participate at a community health clinic as access to apartment complexes for recruitment purposes was not possible due to building security. All households, regardless of location, were given compensation for their participation in the form of popular household consumable goods such as noodles, candy, and fresh bread worth no more than $10 USD. This was selected in consultation with local field staff.

### Sample collection

Each participating household was visited by the research team on two consecutive days. During the first day, Mongolian team members explained the study and obtained informed written consent from an adult household member. A household survey was then administered to this adult by a trained field team member covering topics related to water, sanitation, and hygiene, animal contact, food safety, diarrheal disease, and zoonotic risk perceptions. The survey was initially written in English and translated into Mongolian by local staff (S1 File)

After the survey was completed, a household member was asked to fill a sterile 50mL tube (Falcon 50mL Conical Centrifuge Tubes, Fisher Scientific) with household drinking water and then, following permission, a field team member placed a commercial sticky fly strip ribbon inside the ger near the stove and food prep area. The household was provided with stool containers, gloves, aluminum pans, and instructions for how to safely and correctly collect approximately 5g of stool from up to two household members, to be decided at their discretion [51]. The instructions were written in Mongolian and contained illustrations created by the research team (available by request). Global Positioning System (GPS) data points were recorded for the household using a handheld device (eTrex 10 GPS Navigator, Garmin). Finally, fresh animal stool samples were collected from the ground surrounding the ger from up to five different animal species. Approximately 24 hours later, the ger was revisited and the human stool was gathered and labeled as adult/child and male/female per communication with the household. Adults were considered 18 years or more. Fly strips were examined and up to 20 flies were removed with sterile forceps cleaned with 70% alcohol and air dried between households to prevent cross-contamination [13,52]. Fly pools were placed into a sterile container for each household they were found. Date and time of sample collection were recorded.

For apartment households, the consent and household survey were administered at the community clinic. At the completion of the survey, each participant received a fly strip, human stool containers for households without a toilet or “hats” for toilet stool collection (Standard Speci-Pan, Specimen Collector, Medline), gloves, instructions in Mongolian on safe stool collection for either sanitation method, and animal waste containers if the household indicated they owned a domestic animal. At an agreed upon pick up time for the following day, participants contacted field staff with verbal directions to a pickup address. GPS points were recorded for the overall apartment or housing complex where the participant lived.

### Sample preparation

All water samples were stored at room temperature or -4° C until transported to the Institute of Veterinary Medicine (IVM) for processing. Stool samples (human and animal) were stored at room temperature in 70% alcohol until taken to the IVM laboratory for processing [53–56]. Flies were kept at room temperature or euthanized for several hours in a freezer, until they could be stored at the IVM laboratory for identification using a taxonomic guide to the family level of *Muscidae*, *Calliphoridae*, and *Sarcophagidae* and processed for further analysis [57–60].

Before DNA extraction, stool samples first underwent several steps in preparation. Five grams of stool stored in 70% alcohol was transferred into 15mL tubes (Falcon 15mL Conical Centrifuge Tubes, Fisher Scientific). Using a modified technique described by Hong et al., these tubes were then centrifuged at 2,000 rpm for three minutes followed by the removal of the preservative supernatant [40]. Samples were then resuspended by filling the 15mL tube with phosphate buffered saline (1 X PBS). Centrifugation was repeated at 3,000 rpm for five minutes, with the supernatant again decanted. After the initial phase, the resulting pellet was moved into 1.5ml tubes and stored at -4° C before undergoing the second step.

Fly samples were prepared by adding 5mL of PBS to each 15mL tube containing one fly pool, modified from Clavel et al. [61]. Each tube was then gently rocked (by hand) for two minutes to dislodge occysts from exterior exoskeleton of the fly [52,62–65]. Next, a disinfected plastic rod (chopstick) was inserted into the 15mL tube to macerate the flies for one minute [13,52,58,60–61,63–66]. Then the 15mL tube of homogenized flies were centrifuged at 2,000 rpm for five minutes [62–63]. The liquid supernatant was poured off and the remaining pellet was placed in a clean 2mL tube before the next round of processing [58,16,67].

Household water samples were centrifuged at 4,000 rpm for 15 minutes with a counterweight when needed as modified from Shanan et. al. [68]. Liquid was then carefully poured off the top to leave approximately 0.5mL from the bottom of the tube. Using a clean, wooden stick materials from the bottom were resuspended into the remaining liquid. This liquid containing debris was removed with a clean pipette and place in a new, sterile 2mL tube.

For the next step in processing, the stool, water, and fly samples were placed in a 16 place beaker rack (Beaker Buddy 16 Place, Electron Microscopy Sciences). The secured samples then underwent 15 freeze-thaw cycles wherein the samples were placed in liquid nitrogen for one minute then directly thawed in a water bath of 65° C for one minute to break the thick walls of the *Cryptosporidium* and *Giardia* cysts [69]. The resulting sample was stored at -4° C until DNA extraction.

### DNA extraction and multiplex real-time PCR

DNA was extracted from human and animal stool samples using TIANamp Stool DNA kits (Tiangen Biotech, Beijing, Cat. No. DP328) and from fly and water samples using TIANamp Genomic DNA kits (Tiangen Biotech, Beijing, Cat. No. DP304) following the manufacturer’s instructions. Extracted DNA was then stored at -80° C until PCR amplification.

Target genes comprised the 18S ribosomal RNA (rRNA) gene for *Giardia duodenalis* and the Cryptosporidium Oocyst Wall Protein (COWP) for *Cryptosporidium spp*. [70,71]. Modified amplification reactions were performed with optimized concentrations for primer and probe sequences described in previous studies [70,71]. Amplification consisted of three minutes at 95°C followed by 40 cycles of 30 seconds at 95°C, 30 seconds at 55°C, and 30 seconds at 72°C then finally 7 minutes at 72°C. Amplification, detection, and data analysis were performed on the iCycler iQ Real-Time Detection System (Bio-Rad, California, USA).

### Data analysis

Statistical analyses were performed using Stata IC 15.0 (StataCorp. 2017, College Station, TX, USA). Descriptive statistics were used to determine the prevalence of pathogens in Mongolian households and samples by province, month, and specimen type. Participants that reported more than one primary source of water or sanitation in the household survey were classified by the least unimproved source indicated. According to the Joint Monitoring Programme for Water Supply, Sanitation and Hygiene (JMP), improved drinking water sources (piped water, boreholes or tubewells, protected springs and dug wells, rainwater that can be collected free of environmental contamination, and bottled or delivered water) are typically safer in terms of their design and construction than unimproved water sources (unprotected springs or dug wells, surface water such as streams and lakes, or rainwater or snow that has been collected after environmental contamination) [72].

Improved sanitation methods safely collect and stores waste to avoid human contact and include flushing or pour toilets connected to a sewage system, septic tanks, pit latrines with concrete slabs and ventilated pit latrines, and composting and/or bio-toilets [72]. Unimproved sanitation methods are hanging latrines or pit latrines without concrete slabs, buckets or other containers, and open defecation [72]. Burying stool was considered an improved sanitation method for this study, although its safety is dependent upon where the waste is buried and at what depth.

An unadjusted bivariate logistic regression model tested for significant correlations between the presence of *Cryptosporidium spp*. and/or the presence of *Giardia spp*. in humans, animals, or the environment of participating rural Mongolian residences and variables of interest associated with their household characteristics, animal contact, and water, sanitation and hygiene behaviors. A combined variable was created to represent households that had a single parasite species or both parasite species present, as the exposure pathways for these pathogens are the same. Variables with significance from the bivariate logistic regression model were included in a multivariate regression model, which also adjusted for household location (ex. rural). Results with p ≤ 0.05 were considered statistically significant. Analyses were conducted using only available data and absent data were assumed to be missing at random.

Geospatial operations were conducted using ArcGIS 10.5.1 (ESRI, Redlands, CA) and R 3.6.1 (www.r-project.org) on all study households with successfully recorded GPS points (n = 245). The aim of our geospatial analysis was to identify regional variability in the risk of parasitized human households while controlling for the presence of parasites in water, flies, and animal specimens, regardless of pathogen.

To perform these analyses we constructed a Bayesian spatial regression model using the R package brms, which makes use of the posterior sampling program Stan [73–74]. Our dependent variable was the binary result of parasite presence in a household member, regardless of parasite type. This was modeled using a logistic probability distribution, with the primary independent variable being a 2-dimensional smoothing function of longitude/latitude coordinate space. We used a Gaussian process (kriging) to create a smooth function of coordinate space. Animal, fly, and water testing were incorporated in the models as fixed linear predictors. We chose minimally informative regularizing prior probability distributions for the linear predictors and the intercept, selecting a mean of 0 and standard deviation of 0.5 to constrain the model from making extreme estimates. To assess the impact of animal, fly, and water contamination, we also ran a model adjusted only for spatial coordinates. We then compared the adjusted and unadjusted models by their Watanabe-Akaike information criterion (WAIC).

After running the model, we predicted its results to the geographic regions surrounding our sampling households. The households loosely fell within five clusters, and these clusters were separated by large areas with no sampling (and sparse populations). Using the minimum bounding geometry tool in ArcGIS we created a convex boundary around each of the five clusters, then expanded each of these boundaries using a 10 km buffer zone. We then generated a dense coordinate grid covering the entire study area and retained those coordinate pairs that fell within the buffer zones. We then predicted the odds estimates from the fit spatial model to the remaining coordinates. Odds were converted to probability using the formula odds / (1 + odds), and expressed as predicted prevalence.

## Results

**Fig 2:**
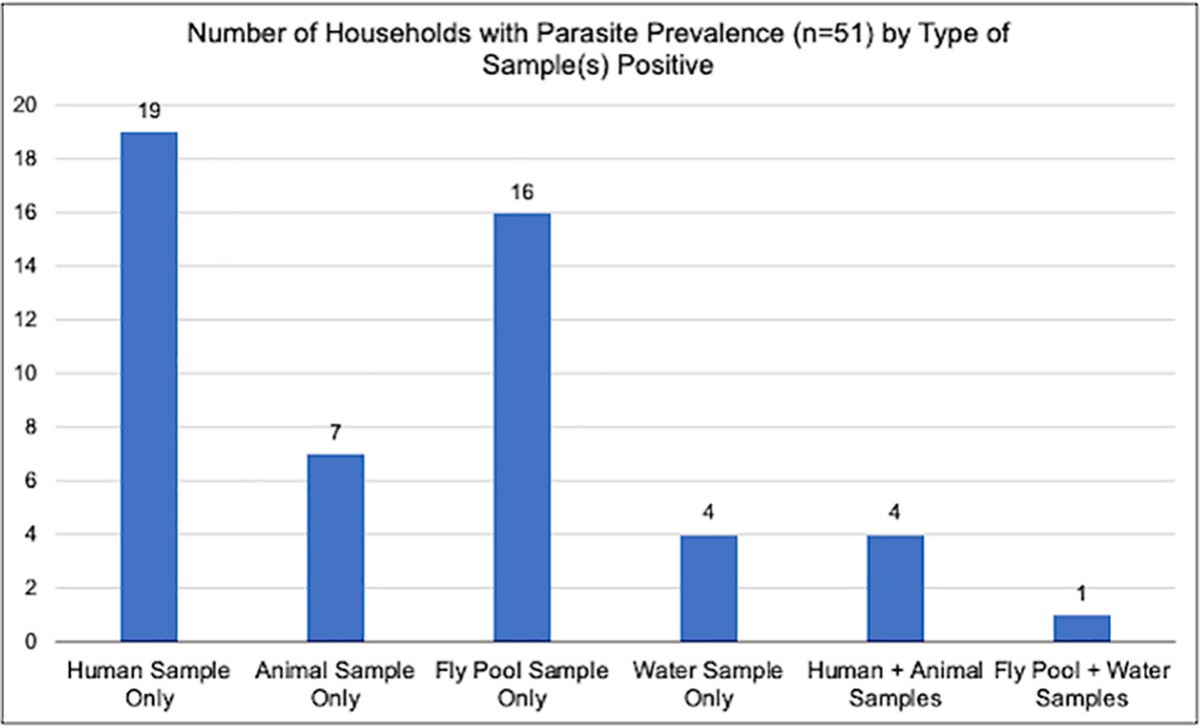
Sample types positive for *Cryptosporidium spp*. and/or *Giardia duodenalis* at each Mongolian household with zoonotic enteric parasite presence.

A total of 250 households participated from rural, peri-urban, and urban sites. Overall, 20% of study households had at least one sample that was positive for *Cryptosporidium spp*. and/or *Giardia duodenalis* (n = 51;Table 1). Household samples included human stool, domestic animal stool, drinking water, and filth fly pools. Polyparasitism occurred in 0.7% of the overall samples tested, while 0.8% of samples were positive for *Cryptosporidium spp*. only and 3.5% positive for *Giardia duodenalis* only. Of the 51 positive households, 19% demonstrated polyparasitism and approximately 8% had one or more ZEPs found in both human and animal samples at the same home site (Fig 2).

**Table 1:** Occurrence of *Cryptosporidium spp*. and *Giardia duodenalis* in human, animal, water, and fly samples (n = 1,354) collected at Mongolian study households (n = 250).

Variable	Study households n	Total positive households n (%)	Samples n	Number of positive samples n			Total positive samples n (%)
				*Crypto spp*.	*Giardia duodenalis*	*Crypto spp*. & *Giardia*	
**Total Households**							
Households	250	51 (20.4)	1,354	11	47	10	68 (5.0)
**Household Location**							
Selenge	50	13 (26.0)	347	1	15	9	25 (7.2)
Zavkhan	50	14 (28.0)	359	3	16	0	19 (5.3)
Dundgobi	50	10 (20.0)	330	1	8	1	10 (3.0)
Peri-Urban Ulaanbaatar	50	7 (14.0)	189	1	6	0	7 (3.7)
Ulaanbaatar City	50	7 (14.0)	129	5	2	0	7 (5.4)
**Sampling Month**							
April	25	3 (12.0)	165	0	1	3	4 (2.4)
May	25	7 (28.0)	184	0	11	0	11 (6.0)
June	50	9 (18.0)	273	1	8	0	9 (3.3)
August	25	10 (40.0)	182	1	14	6	21(11.5)
September	50	13 (26.0)	340	3	10	1	14 (4.1)
October	75	9 (12.0)	210	6	3	0	9 (4.3)
**Household Member**			419				27 (6.4)
Adult Female		10 (19.6)	153	4	4	2	10 (6.5)
Adult Male		7 (13.7)	103	0	5	2	7 (6.8)
Child Female		6 (11.8)	73	0	4	2	6 (8.2)
Child Male		4 (7.8)	89	2	1	1	4 (4.5)
**Domestic Animal**			570				19 (3.3)
Sheep/Goat	226	6 (11.8)	226	0	6	0	6 (2.7)
Cow	130	2 (3.9)	130	0	1	0	1 (0.8)
Horse	108	8 (15.7)	108	0	8	1	9 (8.3)
Dog	94	3 (5.9)	94	0	2	1	3 (3.2)
Other**	11	0	11	0	0	0	0 (0)
Camel	1	0	1	0	0	0	0 (0)
Cat	0	0	0	0	0	0	0 (0)
**Drinking Water**	250	5 (9.8)	250	5	0	0	5 (2.0)
Fly Pools***	115	17 (33.3)	115	0	16	1	17 (14.8)

*When more than one parasite was present in the same samples, the sample was reported to have both *Crypto*. *spp*. and *Giardia duodenalis*

**Other samples collected at the household included rodent, yak, and marmot

***Fly families in pools included Muscidae, Calliphoridae, and Sarcophagidae.

Most positive households were in the rural provinces of Zavkhan (28%) and Selenge (26%). Rural sites also provided more samples per household as compared to the peri-urban and urban sites as they engaged in herding and other animal husbandry operations. Overall prevalence varied by province, with 7% of all Selenge household samples testing positive for *Cryptosporidium spp*. and/or *Giardia duodenalis*.

Parasite prevalence among the household samples was most commonly detected during the warmer months, particularly in August, wherein 11.5% of the collected household samples were positive. All types of household members, regardless of age or sex, were represented among the positive human stool samples provided by the study households. However, the highest parasite prevalence was discovered in female children (8.2%). Among households with either *Cryptosporidium spp*. and/or *Giardia duodenalis*, 19.6% had at least one positive sample occur in an adult female.

The overall prevalence among all domestic animal stool samples was 3.3%. The animal species with the highest zoonotic enteric prevalence was horses (8.3%), followed by dogs (3.2%), and finally sheep/goats (2.7%). Of the positive households, 15.7% demonstrated *Giardia duodenalis* or polyparasitism with *Cryptosporidium spp*. in a horse. Drinking water samples from five study households were positive for *Cryptosporidium spp*. only (2%). Of the filth flies that were available to be collected and pooled in households, 14.8% were positive for one or both parasite species. Among households with any positive sample, 33.3% had a fly pool with *Giardia duodenalis* or polyparasitism with *Cryptosporidium spp*.

The presence of *Cryptosporidium spp*. and/or *Giardia duodenalis* was highest in rural households (25%) as compared to urban and peri-urban households (both 14%; Table 2). Parasite prevalence was more common among households that did not have electricity but instead relied on solar power (25%), as well as households that utilized wood and biofuel (i.e. animal dung) for their main fuel source (25%). This was also the case for households that reported use of animal manure or compost for additional purposes (25%).

**Table 2:** Characteristics of Mongolian Households with Zoonotic Enteric Parasite Presence (n = 51) and Without (n = 199).

Household Characteristic	Households without Parasite Presence n(%)	Households with Parasite* Presence n(%)	Total Households n
All Households	199 (79.6)	51 (20.4)	250
Location			
Urban	43 (86)	7 (14)	50
Peri-Urban	43 (86)	7 (14)	50
Rural	113 (75.3)	37(24.7)	150
Number of Residents			
1–2	35 (85.4)	6 (14.6)	41
3–5	116 (77.9)	33 (22.1)	149
6–8	44 (80)	11 (20)	55
>8	4 (80)	1 (20)	5
Children ≥ 5 Years			
Yes	69 (84.2)	13 (15.8)	82
No	130 (77.4)	38 (22.6)	168
Electricity (n = 249)			
Yes	85 (85.9)	14 (14.1)	99
No	113 (75.3)	37 (24.7)	150
Solar Power (n = 249)			
Yes	110 (74.8)	37 (25.2)	147
No	88 (86.3)	14 (13.7)	102
Main Fuel Source (n = 249)			
Electricity	68 (87.2)	10 (12.8)	78
Propane	6 (100)	0 (0)	6
Wood/Biofuel	119 (74.8)	40 (25.2)	159
Coal	5 (83.3)	1 (16.7)	6
Use Compost/Manure (n = 184)			
Yes	117 (75.5)	38 (24.5)	155
No	26 (89.7)	3 (10.3)	29
Own the Following Assets			
Refrigerator (n = 249)	73 (79.4)	19 (20.6)	92
Tractor (n = 249)	0 (0)	2 (100)	2
Animal-Drawn Cart (n = 249)	1 (20)	4 (80)	5
Car/Truck	104 (80)	26 (20)	130
Motorcycle	83 (78.3)	23 (21.7)	106
Bicycle	7 (77.8)	2 (22.2)	9
Radio	26 (89.7)	3 (10.3)	29
Mobile Phone	192 (79)	51 (21)	243
Computer	35 (85.4)	6 (14.6)	41
Television	170 (79.8)	43 (20.2)	213
Bank Account	190 (78.8)	51 (21.2)	241
Domestic Animal Presence/Ownership			
Yes	144 (76.6)	44 (23.4)	188
No	55 (88.7)	7 (11.3)	62
Presence/Ownership of the Following Animal(s)			
Dog	127 (76.1)	40 (23.9)	167
Cat	15 (75)	5 (25)	20
Horse	101 (74.3)	35 (25.7)	136
Sheep	113 (75.8)	36 (24.2)	149
Goat	114 (77)	34 (23)	148
Camel	3 (75)	1 (25)	4
Cow	105 (76.6)	32 (23.4)	137
Chicken	1 (100)	0	1
Other animal(s) Not Listed	28 (77.8)	8 (22.2)	36
Think **Humans** Can Give Disease or Illness to Animals (n = 249)			
Yes	18 (85.7)	3 (14.3)	21
No	117 (81.8)	26 (18.2)	143
Unsure/Don’t Know	63 (74.1)	22 (25.9)	85
Think **Animals** Can Give Disease or Illness to Humans			
Yes	162 (78.3)	45 (21.7)	207
No	18 (85.7)	3 (14.3)	21
Unsure/Don’t Know	19 (86.4)	3 (13.6)	22

*Positive for *Cryptosporidium spp*., *Giardia duodenalis*, or both zoonotic enteric parasites

Regardless of parasite presence, most households reported owning a mobile phone (97%), television (85%) and having access to a bank account (96%). Among the households in which one or more zoonotic enteric parasites were found, 23% owned domestic animals or domestic animals were present at the site during sampling. Of the households that tested positive, a range of animal species were owned or present, such as dog (24%), horse (26%), sheep (24%), goat (23%), and cow (23%). When asked about zoonotic or reverse zoonotic disease transmission, most survey respondents said that animals can give illness to humans (83%) but less believed humans can give illness to animals (8%). Among the households with parasite presence, only 22% of survey respondents recognized the risk of zoonotic disease transmission.

In total, 36% of the study households (n = 91) reported using an improved source for their primary drinking water, as defined by the WHO/UNICEF Joint Monitoring Programme for Water Supply, Sanitation and Hygiene (JMP) (Table 3) [72]. However, only 28% of the rural households had access to an improved drinking water source. Of households that used unimproved water sources, 43% were positive for one or both parasites. Boiling water before consumption was the common method for drinking water treatment. Still, 92% of the households with parasite presence reported to boil their water before drinking it.

**Table 3:** Water, Sanitation and Hygiene Access and Behaviors at Rural (n = 150), Peri-Urban (n = 50), and Urban (n = 50) Mongolian Study Households and Households with Zoonotic Enteric Parasite Prevalence (n = 51).

WASH Factor	Households				
	Rural n(%)	Peri-Urban n(%)	Urban n(%)	Parasite* Presence n(%)	Total N
**Main Drinking Water Source**					
Improved	42 (28.0)	46 (92.0)	3 (6.0)	11 (21.6)	91
Private Well	2 (1.3)	6 (12.0)	0 (0)	3 (5.9)	8
Shared Well**	2 (1.3)	0 (0)	0 (0)	2 (3.9)	2
*Soum* Center Well**	17 (11.3)	0 (0)	0 (0)	0 (0)	17
Piped Water	21 (14.0)	23 (46.0)	0 (0)	4 (7.8)	44
Tanker Truck	0 (0)	17 (34.0)	0 (0)	2 (3.9)	17
Rainwater	0 (0)	0 (0)	0 (0)	0 (0)	0
Bottled Water	0 (0)	0 (0)	3 (6.0)	0 (0)	3
Unimproved	65 (43.3)	0 (0)	0 (0)	22 (43.1)	65
Lake, River or Stream	49 (32.7)	0 (0)	0 (0)	17 (33.3)	49
Melted Snow***	16 (10.7)	0 (0)	0 (0)	5 (9.8)	16
Other/Source Not Listed	43 (28.7)	4 (8.0)	47 (94.0)	18 (35.3)	94
**Water Treatment Before Use**					
Boil It	123 (82.0)	45 (90.0)	47 (94.0)	47 (92.2)	215
Filter It	3 (2.0)	0 (0)	3 (6.0)	0 (0)	6
Drink Directly From Source	24 (16.0)	5 (10.0)	0 (0)	4 (7.8)	29
**Main Sanitation Source**					
Improved	44 (29.3)	8 (16.0)	49 (98.0)	20 (39.2)	101
Flush Toilet	0 (0)	3 (6.0)	49 (98.0)	7 (13.7)	52
Pit Latrine with Slab	0 (0)	1 (2.0)	0 (0)	0 (0)	1
Bury in Hole	44 (29.3)	3 (6.0)	0 (0)	13 (25.5)	47
Compost or Biotoilet	0 (0)	1 (2.0)	0 (0)	0 (0)	1
Unimproved	105 (70.0)	41 (82.0)	0 (0)	31 (60.8)	146
Pit Latrine No Slab	31 (20.7)	40 (80.0)	0 (0)	13 (25.5)	71
Bucket or Container	0 (0)	0 (0)	0 (0)	0 (0)	0
Open Defecation	74 (49.3)	1 (2.0)	0 (0)	18 (35.3)	75
Other/Sanitation Not Listed	1 (0.7)	1 (2.0)	1 (2.0)	0 (0)	3
**Sink or Handwashing Area (n = 249)**					
Yes	56 (37.6)	41 (82.0)	50 (100)	33 (64.7)	147
No	93 (62.4)	9 (18.0)	0 (0)	18 (35.3)	102
**If Yes, Location (n = 147)**					147
Inside	19 (33.9)	30 (73.1)	50 (100)	17 (51.5)	99
Outside	37 (66.1)	11 (26.8)	0 (0)	16 (48.5)	48
**Handwashing Events**					
In the Morning	140 (93.3)	39 (78.0)	41 (82.0)	45 (88.2)	220
Before Cooking	57 (38.0)	26 (52.0)	32 (64.0)	27 (52.9)	115
Before Eating	53 (35.3)	17 (34.0)	40 (80.0)	17 (33.3)	110
After Urination or Defecation	35 (23.3)	26 (52.0)	44 (88.0)	26 (51.0)	105
After Handling Animals	117 (78.0)	16 (32.0)	3 (6.0)	33 (64.7)	136
Other Times Not Listed	35 (23.3)	13 (26.0)	18 (36.0)	14 (27.5)	66
Never Wash Hands	0 (0)	0 (0)	0 (0)	0 (0)	0

*Positive for *Cryptosporidium spp*., *Giardia duodenalis*, or both zoonotic enteric parasites

**Many respondents wrote in that they used a shared public water well located in the center of each rural soum

***Melted snow can be classified as an improved drinking water source if the collected snow has remained free of environmental contamination such as human or animal waste

Most rural households reported their drinking water primarily came from an unimproved source (43%), such as directly obtaining the water from lakes, rivers or streams (33%) or from melting snow (11%). In some situations, melted snow can be an improved method for procuring safe drinking water. However, observational analysis found the snow surrounding rural households to be contaminated by livestock and human waste without a catchment system in place to keep the snow separated from environmental pollutants.

Overall, 40% of the study households used an improved sanitation source, again as those outlined by the WHO/UNICEF JMP [72]. Yet within the rural households, 70% used unimproved sources of sanitation management, including 49% of rural households who reported practicing open defecation. Of the study households that reported unimproved sanitation services, 61% were positive for one or more zoonotic enteric parasites.

Sink and/or handwashing site availability varied across rural (38%), peri-urban (82%), and urban study households (100%). Of those households that reported having a designated spot for washing hands, 67% were located inside. Yet, of the rural households with handwashing sites, 66% were located outside. Popular self-reported handwashing events of the study households included in the morning (88%), after handling animals (54%), before cooking (46%), before eating (44%), and after urination/defecation (42%).

An analysis of the risk factors identified by the household survey are presented (Table 4). In the bivariate logistic regression model, there were greater odds of parasite presence when a household owned domestic animals (OR 2.40; CI 1.02–5.65; p = 0.05) or was located in a rural area (OR 0.50; CI 0.25–0.98; p = 0.04). But the odds of parasite presence were lower when the household used of an improved drinking water source (OR 0.27; CI 0.12–0.61; p = < 0.01) or had an indoor handwashing site (OR 0.41; CI 0.19–0.92; p = 0.03), Use of improved sanitation methods and practicing open defecation were not associated with household zoonotic parasite presence. In the multivariate model, household use of an improved drinking water source was the only factor that remained statistically significant in relation to *Cryptosporidium spp*. and/or *Giardia duodenalis* presence (OR 0.16; CI 0.04–0.68; p = 0.01). The addition of more explanatory variables in the multivariate model likely contributed to variance in the dependent variable and may have reduced statistical contribution of the previously significant variables from the bivariate model.

**Table 4:** Bivariate and multivariate analysis of the association between household risk factors and the presence of *Cryptosporidium spp*. and/or *Giardia duodenalis* in humans, animals, and the environment.

Variable	Unadj. Bivariate Regression			Adj. Multivariate Regression		
	OR (95% CI)	Std. Err.	*P* value	aOR (95% CI)	Std. Err.	*P* value
**Household Drinking Water**						
Use Improved Water Source	0.27 (0.12–0.61)	0.11	<0.01^b^	0.16 (0.04–0.68)	0.12	0.01^c^
Water Treatment^a^ Prior to Use	1.69 (0.56–5.09)	0.95	0.35			
**Hygiene Behaviors**						
Sink or Hand Washing Site	1.35 (0.71–2.56)	0.44	0.36			
Site is indoors	0.41 (0.19–0.92)	0.17	0.03^b^	1.12 (0.38–3.30)	0.62	0.83
Reported Hand Washing						
In the Morning	1.03 (0.40–2.67)	0.50	0.95			
Before Cooking	1.42 (0.77–2.63)	0.45	0.27			
Before Eating	0.57 (0.30–1.09)	0.19	0.09			
Before Feeding Child	0.72 (0.24–2.20)	0.41	0.57			
After Using Bathroom	1.58 (0.85–2.93)	0.50	0.15			
After Handling Animals	1.71 (0.90–3.23)	0.56	0.10			
Use Compost/Animal Manure	2.81 (0.81–9.82)	1.79	0.11			
**Household Sanitation**						
Use Improved Sanitation	0.92 (0.49–1.72)	0.30	0.79			
Reported Open Defecation	1.21 (0.62–2.36)	0.41	0.58			
**Animal Factors**						
Household Animal Contact	1.87 (0.87–4.04)	0.73	0.11			
Household Animal Ownership	2.40 (1.02–5.65)	1.05	0.05^b^	2.73 (0.74–10.05)	1.82	0.13
**Location**						
Rural Site	0.50 (0.25–0.98)	0.17	0.04^b^	0.87 (0.25–3.09)	0.56	0.83

^a^Treat drinking water by boiling or filtering

^b^Significant at p ≤ 0.05 in the bivariate analysis

^c^Significant at p ≤ 0.05 in the multivariate analysis.

**Fig 3:**
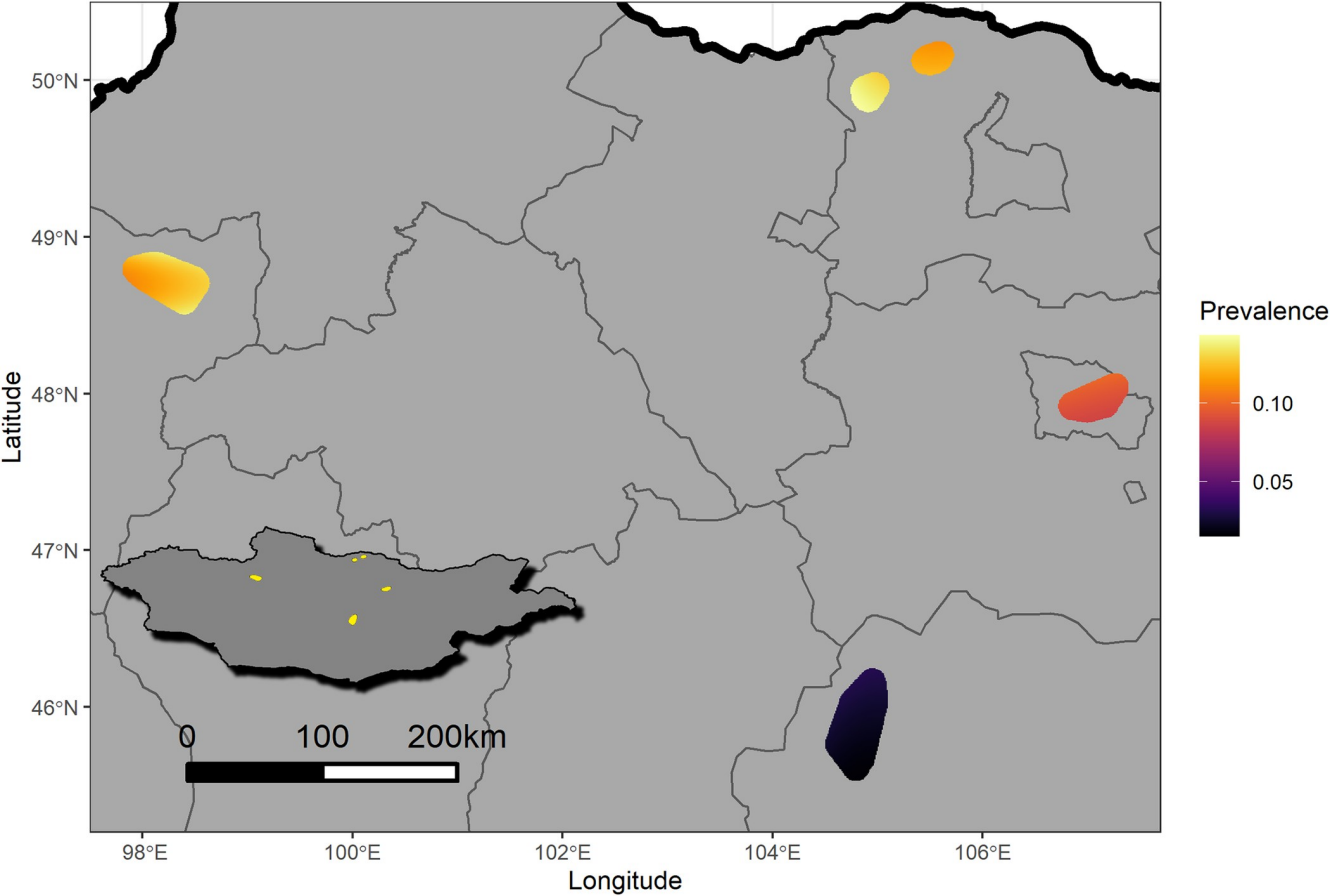
Predicted prevalence that a participating household would have a household member positive for *Cryptosporidium spp*. and/or *Giardia duodenalis*. The probability of a positive specimen was modeled as a function of coordinate location and the results of animal, fly, and water testing. The fit model was then predicted to a dense grid of coordinate locations in the environs of the sampling sites. The model revealed spatial heterogeneity, with lower prevalence of the two pathogens in the southernmost cluster of samples. Adjustment for nonhuman samples did not blunt the spatial heterogeneity.

The prevalence of human parasitosis was heterogeneous among our sampling sites within Mongolia, with a greater prevalence in more northern sites (Fig 3). The odds ranged from 0.015 in Dundgobi province to 0.14 in Selenge province. This heterogeneity was not attenuated by adjustment for environmental parasite exposure, suggesting that other unmeasured factors may contribute to this geographic pattern.

## Discussion

Our findings in this One Health study on the presence of zoonotic enteric parasites and associated risk factors demonstrate *Cryptosporidium spp*. and *Giardia duodenalis* circulating among people, animals, flies, and drinking water within Mongolia, particularly for rural herding families. Fifty-one households had at least one sample type test positive for a ZEP, several of which either had polyparasitism, positivity among multiple sample types, or both at the same home site. This study demonstrates the importance of addressing the simultaneous presence of zoonotic enteric parasites among humans, animals, and their shared environment.

Inequitable access to improved water, sanitation, and hygiene (WASH) infrastructure was highlighted between our participating urban, peri-urban, and rural Mongolian households. Within our study population, households that use an improved drinking water source were less likely to have a positive zoonotic enteric parasite sample than households without drinking water from an improved source (OR 0.27). In an analysis of outbreaks associated with the waterborne parasites *Cryptosporidium spp*. and *Giardia duodenalis*, researchers found that in 82% of the global outbreaks between 2011–2016, risk factors included untreated drinking water, contaminated water sources, ineffective treatment of water, or contamination during storage or at point-of-use [3]. Obtaining water from a source that has been designated as “improved” does not ensure drinking water safety at the time of use. Several opportunities exist for the introduction of waterborne parasites between a drinking water collection source and the point-of-use [75]. These include fecal contamination of water storage containers, utensils or hands used to divvy out water through human waste, animal waste, or contact with insect or rodent vectors [76,77]. In our study population the majority of participants (88%) indicated that they used some form or water treatment before use but still five drinking water samples were positive for *Cryptosporidium spp*. Access to an indoor handwashing site or sink was also a protective factor against parasite prevalence without our study households. Yet this may be an indicator of a household’s socio-economic status or education level. Reliable, available, affordable, and safe water is not only vital for drinking and cooking but is necessary for proper hand hygiene that has been shown to reduce diarrheal disease globally [78]. National efforts towards sustainable and safe water solutions should be a public health priority, with particular attention paid to creating accessible source locations for nomadic herding households and mechanisms and education for storing drinking water cautiously inside the home.

Sanitation within urban households was almost exclusively flush toilets while peri-urban households were typically not supported by municipal waste systems and relied upon unimproved options that do not safely treat or dispose of effluent before it reaches the nearby environment. Rural households also largely utilized unimproved sanitation services with almost half practicing open defecation. Open defecation and unmanaged human waste has been shown to lead to pollution of neighboring water sources and facilitate disease spread to humans and animals [77,79]. The proliferation of waste from humans can facilitate reverse zoonotic transmission of parasites to free-roaming animals who graze in the area [80,81]. However, the human and animal movement associated with nomadic and semi-nomadic herding may help reduce the feces burden surrounding a home site, which could help protect against prolonged ZEP exposure risks [82].

Still, indiscriminate feces from humans and animals are the feeding grounds for many filth fly species that can harbor and transmit *Cryptosporidium spp*., *Giardia duodenalis*, and other diarrheal pathogens [52,83]. After landing or feeding on contaminated excreta or food items, parasite oocysts can be transmitted by intermediate filth fly vectors mechanically on the hairs of their legs or inside their gastrointestinal tracts following ZEP ingestion [58]. Filth fly species, such as the house fly (*Musca domestica*), have been shown to travel distances of up to seven kilometers by flight alone and up to 20 kilometers if utilizing transportation such as a car, boat, or animal [84,85]. Findings from this study indicate that filth flies are important vectors of *Giardia duodenalis* and *Cryptosporidium spp*., within Mongolian living spaces.

The majority of households with a positive sample were herders who are located in pastoral provinces. This study found that living at a rural site and animal ownership were both associated risk factors for the presence of *Cryptosporidium spp*. and/or *Giardia duodenalis* at a household. However, this association disappeared in the larger multivariate analysis. Mongolian herders and pastoralists have frequent animal contact and utilize animal products for sustenance, building materials, cooking fuel, transportation, protection, clothing, and traditional healing and folklore remedies [33,86]. The type of animal contact rural household members engage may depend upon their sex and/or their age [87]. Within this study population, many tasks related to animal husbandry and livestock care were shared between male and female household members, with certain activities such as a slaughtering and butchering predominantly male chores while cooking and milking animals were female chores [35]. Because of this, transmission routes for zoonotic enteric parasite exposures will vary among household members and should be addressed through unique interventions that take these differences into consideration. For example, access to dedicated handwashing sites inside homes may help to reduce foodborne transmission as women can wash their hands after chores, such a milking, prior to meal preparation or child feeding.

Animal contact and movement across steppe pastures in Mongolia are both seasonal activities. Spring calving and lambing, summer horse racing events and festivals such as Naadaam, fall culling practices, and winter illnesses and starvation trials present distinctive risk factors for zoonotic and reverse zoonotic disease transmission [32,35,87,88]. Furthermore, harsh temperatures and weather events such as summer droughts and winter *dzuds* can force herders to move livestock and family gers to new ground and in search of shared food and water sources [88]. Research on temporal and seasonal patterns of diarrheal disease has shown summer months to be more prevalent for cases of zoonotic enteric parasites such as cryptosporidosis, with rates rising with increased temperature and precipitation but also with seasonal exposure risks due to water access and quality and animal and agricultural patterns [89]. In this study, most of the households with parasite presence were sampled in August and September, two of the warmer months of the year in Mongolia. Although the winter months present some of the coldest temperatures felt across the globe with January temperatures ranging from -15°C to -35°C (or 5°F to -31°F), previous research on the viability of infectious *Cryptosporidium spp*. and/or *Giardia duodenalis* oocysts in environmental water samples have demonstrated the potential for viability and infection capabilities at spring melt if the water does not incur freeze-thaw cycles [90,91]. However, the resiliency of parasites decreases as temperatures become more extreme and it is likely that new cases in the warmer months are caused by exposure to animals and humans and not overwintering parasites in water sources [17,91].

Mongolian livestock herds are often devised of horse, cattle, yak, sheep, goats, camels, and in the northern region of the country, reindeer. Animal contact and the purpose of their presence and/or ownership vary by species. For example, dogs are common at rural herding households and within larger peri-urban housing compounds (*khashaas*). They are used for guarding the property or livestock herds, to assist with hunting, and for companionship [37,92]. Each species of animal will utilize their environment in different ways such that their own risk of zoonotic enteric parasites from contaminated water, soil, animal and human waste, flies, and food products may present along distinct exposure pathways, although shared pathways are common and could be increased in highly contaminated spaces. Within the current study, the highest number of samples came from sheep and goats, however the largest parasite prevalence was found in horse samples (8.3%). This is much higher than a study on grazing horses in China which found a positive rates of 2.7% for *Cryptosporidium spp*. and 1.5% for *Giardia duodenalis* [93]. Further research into the mechanisms by which Mongolian horses become infected with *Cryptosporidium spp*. and/or *Giardia duodenalis*, whether it be through environmental, animal, or human exposure, is warranted to both protect the health of the herds as well as their herders.

This study demonstrates that *Cryptosporidium spp*. and/or *Giardia duodenalis* are circulating at Mongolian households across multiple human and animal hosts, environmental reservoirs of flies and water, and in significant relation to risk factors such as source of drinking water, animal ownership, and rural residence. At several sites, parasite presence was found in multiple sample types suggesting transmission is occurring at the household level. However, our model illustrated spatial variability in the probability that a household would have human parasite presence, even after adjusting for parasite presence in animals, drinking water, or flies. Furthermore, the direction of transmission could not be determined in this study. One limitation of this research was that *Cryptosporidium* genotyping was not done and therefore we cannot present the most popular host species associated with each positive sample. There was no distinction between active infection or those who may be asymptomatic carriers, which is an important feature in the detection and prevention of zoonotic disease. A better capture of symptomology and health-seeking behavior data would be helpful. Another limitation of this study was that the household survey relied upon self-reporting assets, characteristics, behaviors, and risk factors which may introduce response and recall bias. Particular animal contact questions related to animal husbandry practices failed to include a distinction on whether household member were employed in that task during the time of sampling, which resulted in almost all herding household reporting engagement in these activities. Future work in this area should specify when the activity last took place and/or enlist an observational component to try and better match herding risk factors with parasite presence.

Moreover, the multistage sampling utilized relied heavily upon convenience methods which has a high likelihood of sampling bias. Sampling was done in rural communities (*baghs)* and among household groups (*khot ails)* that could be reached by four-wheel drive vehicles, revisited the following day, and their district (*soum)* could be located within a one to two day drive from our laboratory base. This excluded hard to access provinces and rural households which could have provided different insight or larger, more representative results. The study authors encourage future complementary One Health research be conducted within these regions for comparison and to better our overall understanding of disease risk and transmission pathways among marginalized groups.

Although most participating households indicated a belief in zoonotic transmission, or that animals can give disease to humans, the majority disagreed that humans can give disease to animals. Education on both zoonotic and reverse zoonotic disease risks would be beneficial within a larger One Health intervention strategy. Rural efforts to promote access to improved drinking water, effective household methods for safe storage and treatment prior to use such as disinfection through chlorination or sunlight or filtration, and indoor handwashing sites can help to prevent zoonotic enteric parasite transmission and subsequent diarrheal disease among herding families [94]. However, it must be coupled with culturally appropriate tactics to prevent transmission to humans from animal contact, to animals from human contact, between household members, between animals, and from contaminated environmental sources such as filth flies. The cultural appropriateness and acceptability of interventions will require input from members of the target community. Rural herding households should be invited stakeholders among larger One Health teams of public health professionals, veterinarians, clinicians, epidemiologists, anthropologists, ecologists, agricultural leaders, and others who have a vested interested in the health, safety, and longevity of Mongolia’s human-animal connection.

## Conclusion

The diarrheal disease-causing zoonotic enteric parasites of *Cryptosporidium spp*. and *Giardia duodenalis* are putting the health of Mongolia’s people, animals and environment at risk. However, the culturally and economically significant practice of keeping livestock and herding throughout Mongolia should not be discouraged but instead made safer for both humans and animals using a One Health approach. Public health and veterinary messages aimed at reducing exposure risks should be designed in tandem with insight from local herding families. Additionally, municipalities and provincial governments should work to improve water, sanitation and hygiene access and safety for all households across rural, peri-urban, and urban areas of Mongolia to improve the health of all residents.

## Supporting information

S1 File Household Survey Instrument. (DOCX)

S2 File Strobe Statement Checklist. (DOCX)
