# Supplementary material for: Zoonotic enteric parasites in Mongolian people, animals, and the environment: Using One Health to address shared pathogens
Source: PLoS Negl Trop Dis. 2021 Jul 8;15(7):e0009543. doi: 10.1371/journal.pntd.0009543 (PMC8266129; doi:10.1371/journal.pntd.0009543)
Supplement: S1 File — (DOCX) [file pntd.0009543.s001.docx]

**HOUSEHOLD SURVEY**

**GPS Coordinates**:________________ **Survey Code: ___________**  UB Apt  UB Ger  Rural

**Interviewer Name**: __________________________ **Date of Interview**: _____/______/________

**Province**: ____________ **Soum**: ____________ **Bag**: ____________ **Area**:______________

| **Respondent Information** |
| --- |
| 1. What is your highest grade completed? ______________  2. Respondent sex:  F  M  3. What is your age? ______________  4. Are you the head of the household?  Yes  No  **If no**, b) What is the occupation of the head of household: _____________________  c) What is the sex of the head of household?  F  M  d) What is the highest grade completed for the head of the household? _____________ |
| **Household Demographics** |
| 5. How many people live at this household? _______  b) How many are children? _______  c) What are the ages of the children? (please list): ______________  6. Housing type:  Ger  House  Apartment  Other: _____________________  7. Do you own your home?  Yes  No  8. Does your household have the following? (mark all that apply)   Electricity  Solar-powered generator  Refrigerator  Tractor  Animal-drawn cart  9. Does anyone in your household own the following? (mark all that apply)   Car/truck  Motorcycle  Bicycle  Computer  Television  Radio   Mobile phone  Bank account  10. What is the household’s main source of heating fuel?   Electricity  Propane  Wood/Biofuel  Coal  Other: ______________ |
| **Water, Sanitation and Hygiene** |
| 11. What is your household’s main source of drinking water?   Individual well  Shared well  Piped water to household/property  Lake/river/stream  Tanker truck  Rainwater  Bottled water  Other (list all): __________________  12. Does your household do any of the following to water before drinking it?   Boil it  Filter it  Other: _________________  Drink it directly  13. Does anyone in the household eat the following foods? (mark all that apply)   Raw or undercooked meat  Raw or unprocessed milk/milk products   Unwashed and uncooked vegetables  Unwashed and uncooked fruit  14. Do you have a sink or hand washing area?  Yes  No  **If yes**, b) Where is it?  Inside  Outside  c) Is there soap?  Yes  No  15. When do you wash your hands? (mark all that apply)   In the morning  Before cooking  Before eating  Before feeding children   After toilet  After handling animals  Other reason:____________  Never wash hands  16. What kind of toilet or sanitation system does your household use?   Flush/pour flush toilet  Pit latrine (with slab)  Pit latrine (no slab)  Compost/biotoilet  Bucket/container  Bury in hole  Open defecation on ground  Other: ____________  17. Do you clean your backyard or outdoor space?  Yes  No  **If yes**, b) How often:  Every day  Every week  Monthly  Other: ____________ |
| **Farm and Animal Husbandry Information** |
| 18. Do you own a farm or agricultural land?  Yes  No  **If yes**, a) For what purpose? (mark all that apply)   Growing grains  Growing vegetables  Growing fruit  Herding/livestock  Other: _________________  b) What is the water source for your farm or agricultural land?   Central piped water  Well  Tank truck  Lake/river  Rainwater  Other: ________  19. Do you have contact with animals?  Yes  No  **If yes**, b) For what purpose? (mark all that apply)   Animal husbandry  Herding  Hunting  Pets/companion animals  Racing   Religion  Other: _________________  None/no animal contact  20. Do you use any compost or livestock manure/waste?  Yes  No  **If yes**, b) What kind? (mark all that apply)   Commercial compost  Livestock waste  Other: ___________  c) Why did you use the compost/manure? (mark all that apply)   For fire  To fertilize crops  As building material  Other: ___________  21. How many animals are present at this household? (mark all that apply)  a) Adult dog(s): ________ Baby dog(s): ________  Owned by household  b) Adult cat(s): ________ Baby cat(s): ________  Owned by household  c) Adult chicken(s): ________ Baby chick(s): ________  Owned by household  d) Adult cattle: ________ Baby calves: ________  Owned by household  e) Adult horse(s): ________ Baby foal(s): ________  Owned by household  f) Adult sheep: ________ Baby sheep: ________  Owned by household  g) Adult goat(s): ________ Baby goat(s): ________  Owned by household  h) Adult camel(s): ________ Baby camel(s): ________  Owned by household  i) Other animals (please list with numbers): _______________________________  **If household owns a dog,** j) Where do you keep your dog?   In the home  Tied up in the yard  Not tied in the yard  Other :_________  k) Do you clean up your dog’s stool/waste?  Yes  No  **If yes**, l) How often?  Each time  Daily  Weekly  Monthly  Other: ________  **If household owns any animals**, m) Do your animals ever get diarrhea?  Yes  No  **If yes**, n) What kind of animal (list all): _________________________________  o) How old were they? (mark all that apply)  Adult  Baby  Both  p) How many times each year? __________  q) What month(s) of year? _________________________  r) Do you call a veterinarian?  Yes  No  s) Did any animal die of diarrhea in the past year?  Yes  No  **If yes** t) How old were they? (mark all that apply)  Adult  Baby  Both  u) What month(s) did the most animals die from diarrhea? _______________  v) How many died? __________  Who is responsible at your household for the following livestock-related activities?  w) Herding animals  F  M  Both  None/doesn’t occur  x) Feeding animals  F  M  Both  None/doesn’t occur  y) Milking livestock  F  M  Both  None/doesn’t occur  z) Treating sick animals  F  M  Both  None/doesn’t occur  aa) Slaughtering livestock  F  M  Both  None/doesn’t occur  bb) Butchering livestock  F  M  Both  None/doesn’t occur  cc) Cooking meat and milk  F  M  Both  None/doesn’t occur  dd) Helping with animal births  F  M  Both  None/doesn’t occur  22. Are any animals allowed inside the home?  Yes  No  No animals at household  **If yes,** b) What kind of animal(s)? (list all) ____________________________  c) Why are they allowed inside? (list all): ____________________________ |
| **Human Diarrheal Symptom History** |
| 23. Did anyone in your household have diarrhea in last two weeks?  Yes  No  Don’t remember  **If yes**, b) What was the stool like?  Watery  Bloody  Mucus-like  Don’t remember  c) Were there other symptoms?  Abdominal cramps or pain  Other___________________  d) How often did the diarrhea occur?   Once daily  Twice or more daily  Every few days  Don’t remember  Other: ______  e) Was the household member who had diarrhea an adult, child, or both?   Adult only  Child only  Both an adult and child had diarrhea at the same time  f) Did the person(s) ill from diarrhea attend the hospital?  Yes  No  g) If you provided home care/treatment, what did you do? (mark all that apply)   Did nothing  Rest  Gave lots of fluids  Gave oral rehydration (sugar/salt mixture)   Gave zinc  Gave antibiotics or other drugs  Gave other treatment (list:) ___________ |
| **Risk Perception and Knowledge** |
| 24. Do you think that humans can give disease or illness to animals?  Yes  No  Unsure  25. Do you think that animals can give disease or illness to people?  Yes  No  Unsure  26. Do you think that contact with animals present a health risk to humans?  Yes  No  Unsure  **If yes**, b) What is the risk? (mark all that apply)   Bites and scratches  Diarrheal illness  Ticks, fleas, & mites  Diseases in stool   Diseases in blood, meat & tissues  Animals are unclean  Other: ________________  27. What should people do to be safe around animals? (mark all that apply)   Avoid contact  Vaccinate them  Remove animal waste  Keep outdoors  Other: ________________ |
